# Potential of Carbon Aerogels in Energy: Design, Characteristics, and Applications

**DOI:** 10.3390/gels10060389

**Published:** 2024-06-07

**Authors:** Gazi A. K. M. Rafiqul Bari, Jae-Ho Jeong

**Affiliations:** School of Mechanical Smart and Industrial Engineering, Gachon University, 1342 Seongnam-daero, Sujeong-gu, Seongnam-si 13120, Gyeonggi-do, Republic of Korea; grafiqulbari@gachon.ac.kr

**Keywords:** carbon, aerogels, energy, batteries, microporous, hierarchical, gels, porosity

## Abstract

In energy applications, the use of materials with hierarchical porous structures and large surface areas is essential for efficient charge storage. These structures facilitate rapid electron and ion transport, resulting in high power density and quick charge/discharge capabilities. Carbon-based materials are extensively utilized due to their tunable properties, including pore sizes ranging from ultra- to macropores and surface polarity. Incorporating heteroatoms such as nitrogen, oxygen, sulfur, phosphorus, and boron modifies the carbon structure, enhancing electrocatalytic properties and overall performance. A hierarchical pore structure is necessary for optimal performance, as it ensures efficient access to the material’s core. The microstructure of carbon materials significantly impacts energy storage, with factors like polyaromatic condensation, crystallite structure, and interlayer distance playing crucial roles. Carbon aerogels, derived from the carbonization of organic gels, feature a sponge-like structure with large surface area and high porosity, making them suitable for energy storage. Their open pore structure supports fast ion transfer, leading to high energy and power densities. Challenges include maintaining mechanical or structural integrity, multifunctional features, and scalability. This review provides an overview of the current progress in carbon-based aerogels for energy applications, discussing their properties, development strategies, and limitations, and offering significant guidance for future research requirements.

## 1. Introduction

In energy applications, the utilization of materials with a hierarchical porous structure and large surface area is crucial for efficient charge storage. An open porous structure facilitates rapid electron and ion transport, providing high power density and swift charge and discharge capabilities. Furthermore, engineering specific porosities is essential to maintain a consistent surface area under varying conditions. Identifying appropriate porosities and morphological structures for different applications is a significant factor in optimizing performance [1,2,3].

Carbon-based materials are extensively used in energy applications. The performance of these materials in specific applications depends on various properties, such as pore sizes ranging from ultra-, super-, micro-, and meso- to macropores and surface polarity. Incorporating heteroatoms such as nitrogen (N), oxygen (O), sulfur (S), phosphorus (P), and boron (B) into the carbon structure modifies the surface properties of the carbon walls, significantly influencing the electrocatalytic properties and overall performance of energy devices [4,5,6]. Even carbon materials with a high micropore content may exhibit poor catalytic activity if there are insufficient accessible diffusion paths to the core of the structure. Therefore, a hierarchical structure with efficiently accessible pores ranging from ultra- to macropores is necessary [7]. 

Finding an optimal combination of pore sizes for specific applications remains a challenge. An optimal design should maximize volume utilization and ensure fast, efficient access to the core of the structure. The microstructure of carbon materials significantly impacts energy storage performance. It is essential to consider factors such as the degree of polyaromatic condensation, lateral size, crystallite structure, interlayer distance, R-value (the portion of graphitic crystallites), and defects in carbon materials for various applications [8,9]. Designing a combination of chemical and structural properties requires careful consideration, as heteroatom insertion can reduce the degree of polyaromatic condensation and potentially destroy the pore structure, affecting long-term operational flexibility. Crystalline phases may enhance stability over long life spans [10]. 

Carbon aerogels, derived from the carbonization of organic gel products, are materials with an open porous sponge-like structure in which the liquid component of a gel has been replaced with air [11,12]. These organic gels are prepared from various organic precursors, such as resorcinol, cellulose, formaldehyde, and lignin, using the sol–gel method [13,14]. Carbon aerogels provide a 3D network structure with large surface area and high porosity, along with a stable configuration, making them suitable for energy storage applications. The gel formation process occurs under specific conditions, followed by a critical drying process where the liquid phases inside the gel’s pores are removed without collapsing the structure. The advantages of carbon aerogels include their large surface area and high porosity, which enable them to store a considerable amount of electrical charge, resulting in high energy density. The open pore structure of carbon aerogels favors fast ion transfer, leading to quick charge/discharge rates and high power density. In designing nano- and microstructure strategies, a proper balance or connectivity of surface area and pore size is essential for optimizing the properties of aerogel materials [15,16,17]. However, mechanical integrity is a concern for carbon aerogels, as their lightweight, porous, and low-density nature makes them susceptible to collapse under mechanical stress. Scalability and industrial competitiveness are also concerns due to the complexities of the process. Additionally, single-carbon aerogel materials often cannot provide multifunctionality due to their limited features. This review provides an overview of the current progress and utilization of carbon-based aerogels in energy applications. It discusses their properties, development strategies, and the limitations of current research trends, offering significant guidance for future research requirements.

## 2. Fundamentals of Carbon

Carbon preparation utilizes various technical processes, such as sol–gel, hydrothermal, and pyrolysis at different temperature ranges. The sol–gel and hydrothermal processes provide various initial intermediates, offering opportunities to tune the properties of the carbon, which are later refined through thermal treatments [18]. Pyrolysis, a thermomechanical conversion strategy, decomposes materials into solid, liquid, and gas phases as char, oil, and syngas, respectively, through thermal treatment. 

**Figure 1:**
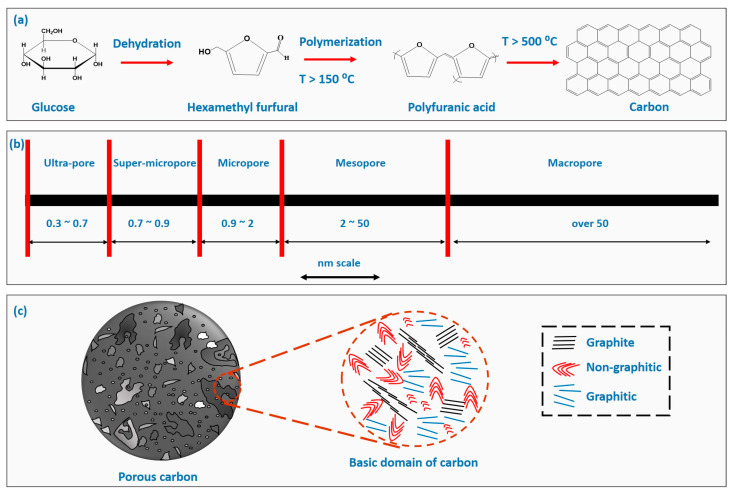
(**a**) Schematic representation of carbonization from the organic precursors, (**b**) schematic of the defined pore sizes, and (**c**) schematic of porous carbon (ultra- to macropores) and basic domain of the carbon.

The carbonization of the organic precursor proceeds through dehydration, followed by intramolecular condensation and decarboxylation, resulting in the formation of various intermediates. Subsequent polymerization of these intermediates occurs through thermal treatment (Figure 1). At high temperatures, polycondensation provides both the amorphous and graphitic phases [19,20]. The choice of precursor, such as biomass-based precursors, organic precursors, or supramolecular precursors, fundamentally affects the morphological, textural, structural, and microstructural properties, which significantly influence performance in specific applications. Additionally, different processing and fabrication conditions greatly impact the final carbon properties [21,22]. 

**Figure 2:**
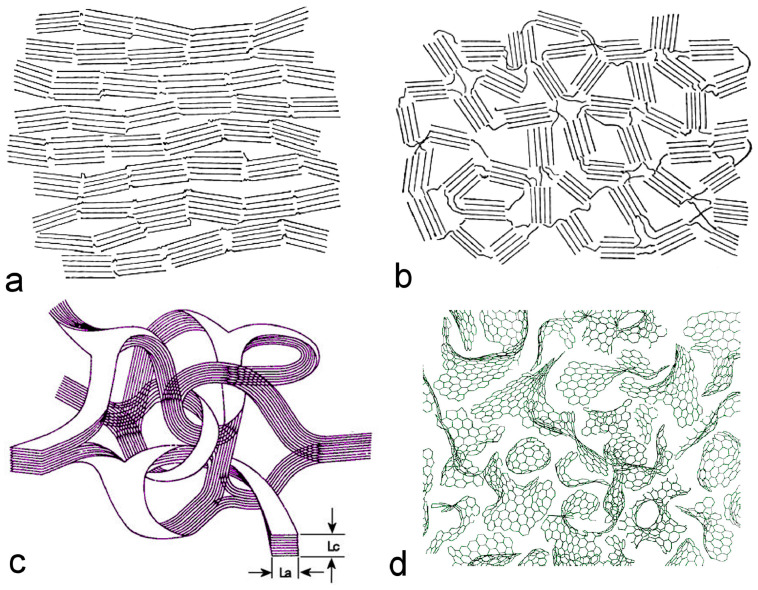
(**a**) The development of mainstream models explaining the relationship between graphitic order and amorphous disorder in glassy carbon (GC) has advanced significantly over time. This progression begins with Franklin’s classification of amorphous carbons into graphitizing and (**b**) non–graphitizing types, (**c**) it further develops through Jenkins’ model, which addresses non–graphitizing GC and associates its crystallinity with La and Lc domains, and (**d**) the evolution reaches its peak with Harris’ model, which portrays non-graphitizing carbons as consisting of intertwined graphitic and fullerene–like structures (adapted with permission from ref. [25], Copyright 2021 Elsevier).

The evolution of carbon structure with temperature leads to multiphase graphitization in non-graphitizing carbon. Analysis of the XRD 002 band deconvoluted peak reveals both crystalline and amorphous structures (Figure 2). The crystalline structure can be further categorized into two types: turbostratic (T–C) and hexagonal graphitic (G–C), observed at 26° 2θ (CuKa radiation), with interplanar spacings of 0.340 nm and 0.335 nm, respectively [23]. GC is considered a potential material in different applications. Initially considered to possess crystallographic and mechanical properties, glassy carbon (GC) exhibits short-range crystalline order, akin to a glass-like fracture [24,25]. GC shares similarities with amorphous carbon, forming at elevated temperatures, characterized by pure sp^2^ hybridization containing three σ and one delocalized π bond per coordination. M. Jenkins et al. regarded GC as comprising randomly oriented, isotropic, and interlocked twisted microfibrils. In the presence of GC, large voids are observed, resulting in a lower material density compared to graphite, approximately ~1.5 vs. 2.3 g cm^−3^. Microcrystalline details describe the microcrystalline length (L_a_) perpendicular and the stacking thickness (L_c_) parallel to the c-axis of the graphite graphene plane, resembling stacked ribbons [25]. The Orleans model delineates graphitization as a two-stage process: in the first stage, stacking order increases, accompanied by the presence of defects in the boundaries with adjacent structural units. Upon thermal treatment, L_a_ disappears, and three-dimensional graphitic order develops concurrently. In the second stage, the wrinkled crystallite layer flattens under the influence of thermal stress. However, in the case of non-graphitizing carbon, this phenomenon does not occur due to the crosslinking of neighboring graphitic fragments, even at high temperatures of 3000 °C during annealing [25].

## 3. Carbon Aerogel Synthesis Strategy

Achieving the optimal properties of carbon gel products hinges on meticulous attention to each processing step, as influenced by myriad factors throughout synthesis and fabrication. From the conditions governing the sol–gel process to those dictating drying, carbonization, and activation, each stage offers ample scope to finely tune or engineer material properties from multifaceted viewpoints pertinent to energy materials. This section delves into the fundamental conditions and phenomena underlying carbon gel product development, providing a mechanistic understanding essential for effective optimization.

### 3.1. Gel Production

**Figure 3:**
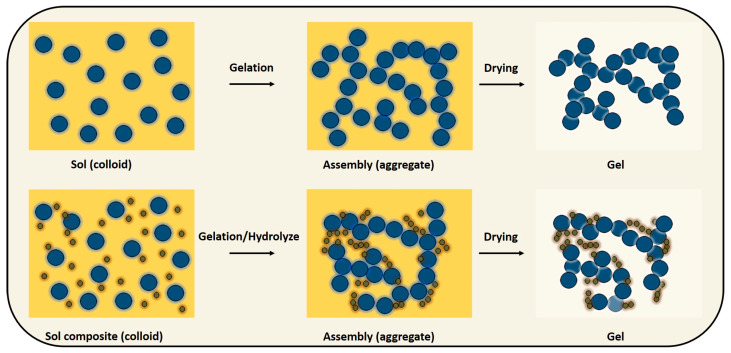
Schematic showing sol–gel synthesis.

The formation of colloidal suspensions or solutions involves the dispersion of precursor compounds, which may be organic, metallic, or metalloid–based, in a solution (Figure 3). This dispersion leads to the creation of a sol, characterized by tiny solid particles dispersed within a continuous liquid medium. The colloidal particles originate from hydrolysis and polymerization reactions. During hydrolysis, the precursor molecules undergo breakdown into smaller particle units, which then undergo further polymerization or interconnection, resulting in the formation of a sol. Under stable reaction conditions, the sol undergoes a transition or gelation process to form a gel, characterized as a biphasic system comprising both solid and liquid phases. The morphological characteristics of the gel involve discrete particles to a continuous polymer network range, representing a complex interplay of structural elements within the material [26,27,28,29].

The ultimate density of gel products is contingent upon the initial density of the reactants within the solution. The relative proportions of reactants, catalyst, and solvent significantly impact the morphological characteristics of the resulting gel products. Lower ratios of reactants to solvent and reactants to catalyst yield smaller particles or pore sizes, reduced compaction, and fewer voids within the gel structure, thereby augmenting the surface area of xerogels. Furthermore, the utilization of acidic or alkaline catalyst solutions and the pH conditions during the application of curing agents exert notable influences on the textural properties of the resultant carbon aerogel products [26,30].

### 3.2. Drying of Gels

**Figure 4:**
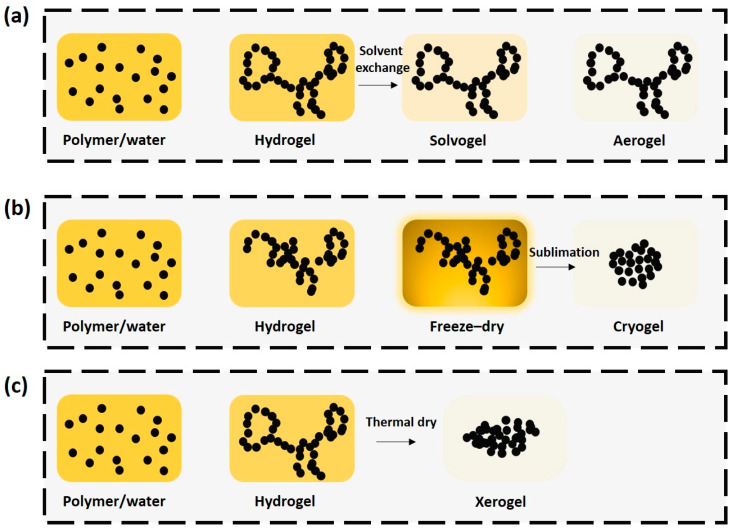
Schematic showing gel drying processes of (**a**) supercritical drying, (**b**) freeze drying, and (**c**) thermal drying.

The engineering of the drying process or conditions is critical for gel production as it significantly influences the properties of the final products. Several drying conditions are employed for gel drying, including ambient drying, supercritical drying, subcritical drying, vacuum evaporation, freeze drying, and convective drying (Figure 4). Gels typically dry under ambient conditions or through simple evaporation, where capillary forces at the liquid–vapor interface cause them to shrink and crack. To mitigate this, drying methods such as supercritical drying, freeze drying, subcritical drying, and microwave drying have been developed to reduce the capillary force at the liquid–vapor interface. As a result, these drying processes help maintain the original porous structure after drying or carbonization. However, methods like supercritical and freeze drying are costly and complex, making them difficult to handle [31].

The subcritical drying process is conducted solely through vacuum drying, requiring no material pretreatment. Wet gels are placed in an unsealed container and maintained at a temperature of approximately 60 °C, while the pressure gradually decreases from 1 bar to 0.1 bar over a period of approximately five days. These time frames can be adjusted with convection drying. The textural properties of the products are influenced by the drying conditions. In the final stage, samples are dried at 150 °C for three days under a pressure of 0.1 bar to ensure complete solvent removal [31,32].

In general, aerogels are typically obtained through supercritical drying. Initially, an aqueous solvent is exchanged with an organic solvent (such as ethanol or acetone) to prepare a solvogel. The gels are then dried under supercritical conditions, commonly utilizing carbon dioxide. During this process, supercritical molecules form a mixture with the organic solvents. The organic solvent replaces the aqueous solvent to create the solvogel, which is prepared at mild pressure and temperature conditions. The supercritical mixture exhibits zero surface tension and preserves the original texture of the gels. Care must be taken to ensure complete removal of water, as incomplete removal may lead to aerogel shrinkage. Particularly for gels with small nodules and pores, solvent removal can be challenging, and capillary forces are enhanced due to the small pore size. Moreover, low-density gels exhibit low resistance to surface tensions, potentially causing integration issues within the skeleton. Despite its superiority in minimizing the shrinkage of meso–/macro–pores, the industrial–scale implementation of this method remains highly costly [33,34,35].

Microwave drying operates under electromagnetic fields through dipole reactions among dielectric molecules. This process yields short drying times but often results in poor textural properties. However, employing high-concentration catalysts can enhance porous properties. Additionally, ultrasonication treatment followed by microwave treatment could improve mesoporous structure. It is noteworthy that ultrasonication does not influence pore size distribution. Ultrasonication treatments lead to the generation of more free radicals on the catalyst, facilitating faster reaction times and yielding larger particles. Microwave drying offers several advantages, such as eliminating the need for external heating, achieving rapid drying rates, preventing hot-spot formation, ensuring uniform material drying, and maintaining low operational costs [36,37].

Freeze-drying methods remove the solvent from wet gels by sublimation after freezing them under low-pressure conditions, resulting in cryogels. The freeze-drying process consists of three steps: freezing the wet gels, sublimating solvents, and desorbing residual solvents from molecules. Consequently, freeze-drying produces large pores in the solid, which are generated by ice crystals and are termed as megaloporosity [37,38].

### 3.3. Carbonization of the Gel

**Figure 5:**
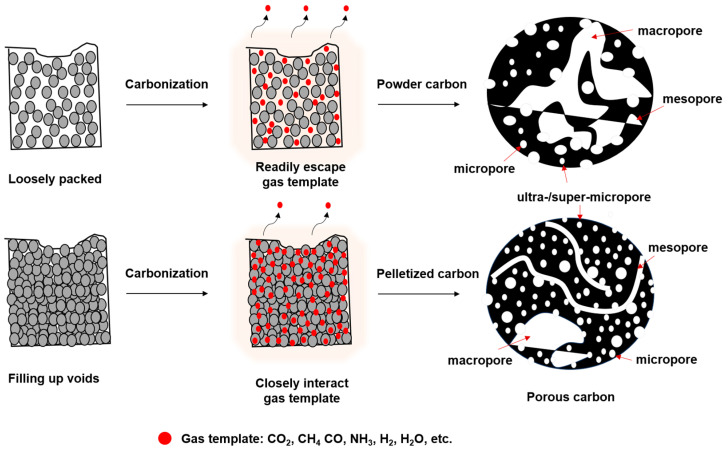
The schematic illustrates the distinct differences in porosity between powder-based carbonization and pelletized-based carbonization. (Adapted with permission from ref. [41], Copyright 2024 Bari et al. MDPI).

Using thermal energy for the thermochemical decomposition of synthesized organic gels at elevated temperatures (400–1000 °C) under continuous inert gas conditions (N_2_, Ar, He) yields carbon gels. The carbonization process lasts for 0.5–5 h, depending on the desired properties or optimization goals related to textural properties, microstructure, and surface chemistry for various applications. Various microstructure properties are considered, including the degree of aromatic condensation, lateral size, stacking height, interlayer distance, defect nature, R-factor, amorphous region, crystalline nature, and turbostratic nature of the carbon. Regarding textural properties, factors such as pore size range (ultra-/super-/micro-/meso-/macropores) and pore volume are carefully adjusted to best suit the intended application and desired properties [11,39,40]. The conditions pertaining to microstructure, textural properties, surface chemistry, or the content of heteroatoms in the carbon gel depend on pyrolysis conditions such as the type of inert gas, gas flow rate, amount of pyrolysis precursors, pyrolysis temperature range, heating rate, pyrolysis time, and volume of the pyrolysis tube. Additionally, whether the pyrolysis is conducted with powder or pelletized precursors is a significant consideration. For powder conditions, factors such as the shape of the container (e.g., boat-shaped, round-shaped) and amount and thickness of the powder need to be taken into account. If pelletized precursors are used, factors such as the amount of organic precursors pelletized, pellet shape, size, thickness, pelletization compression range, and compression time are important (Figure 5). The variability in these conditions leads to the production of carbon gels with distinct properties, highlighting the beauty of research in this field [41].

### 3.4. Activation of the Carbon Gel

Carbon or organic gels require precise tailoring of their pore size and structure to meet the specific criteria of various applications, ensuring an optimal alignment of properties for efficiency. The activation process is conducted on the precursors of the organic or carbon gel, or their intermediary forms, employing various strategic approaches such as chemical activation, physical activation, hard template activation, soft template activation, gas template activation, and metal ion activation. This activation provides the three-dimensional porous structure with either perfect or enhanced surface area and porosity, facilitating ease of mass diffusion or internal access to catalytic active sites for applications related to energy storage and conversion [41,42].

The chemical activation process utilizes acidic (e.g., H_2_SO_4_, HCl, HNO_3_, ZnCl_2_), alkaline (e.g., NaOH, KOH, K_2_CO_3_), and oxidizing agents (e.g., KMnO_4_, H_2_O_2_) directly mixed with precursors, intermediates, or sources and carbonized at different temperature ranges. An optimized ratio of targeted components to activating agent influences the structural, textural, and mechanical properties of the products. The reaction of activating agents with carbon species or volatile groups such as CO, CO_2_, and water, along with spatial positioning and penetration into the internal structure, effectively activates the carbon structure, inducing internal porosity. Upon removal or washing of the activating agents, the internal pore network is revealed [43,44,45].

**Figure 6:**
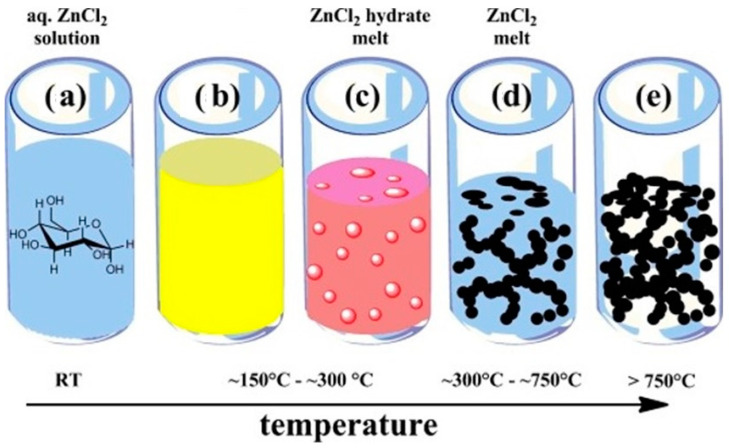
Diagram illustrating the carbonization process of glucose: (**a**) initial solution, (**b**) beginning of low–molecular–weight carbon precursor formation, (**c**) water evaporation and creation of a ZnCl_2_ melt, (**d**) phase separation and development of carbon particles containing ZnCl_2_, and (**e**) ZnCl_2_ evaporation leading to the final porous carbon material (adapted with permission from ref. [47], Copyright 2015 Wiley).

To achieve the desired carbon pore structure in various energy materials for optimal performance, diverse synthesis strategies are employed to engineer the pore architecture. The introduction of effective hierarchical pore morphology, encompassing ultra-/super-/micro-/meso-/macropores, involves employing different activation methods such as chemical activation, physical activation, hard templating, and soft templating approaches [41,42]. Generally, the synthesis of sol–gel carbon products involves utilizing porogens, often considered as a form of soft templating, wherein the degradation of synthesis products yields gaseous templates (e.g., CO_2_, NH_3_, CO, H_2_, H_2_O, CH_4_) at high-temperature carbonization, inducing porosity in the carbon structure. Additionally, to engineer effective micropores, various activation or templating strategies are employed to tailor micropores-to-mesopores within the carbon structure [11,39,46]. These salts can be easily removed from the synthesis products through evaporation or water-washing steps. The Fellinger group, for instance, employs biomass-based carbon precursors (e.g., glucose) with ZnCl_2_ as a salt templating agent to synthesize hierarchically porous heteroatom-doped carbon aerogels for lithium sulfur batteries (LSBs) (Figure 6) [47]. 

The synthesis process involves dispersing low-molecular-weight carbon precursors with salt in an aqueous solution, followed by carbonization at temperatures up to 300 °C, resulting in the separation of carbonaceous particles and the formation of a carbon sol. At temperatures exceeding 300 °C, salt melting and the aggregation of carbon particles lead to the formation of a carbon gel, characterized by interstitial porosity resulting from the presence of porogen phases. Simultaneously, the nanoscopic salt inside the particles acts as salt templating, providing microporosity. Upon reaching temperatures over 750 °C, the salt evaporates, maintaining the highly porous carbon gel foam with a surface area of 1250 m^2^ g^−1^ and a pore volume of 1.2 cm^3^ g^−1^. This extensive porosity facilitates the incorporation of 50 wt.% sulfur as a cathode for LSBs, exhibiting an initial capacity of 1290 mAh g^−1^ in the first cycle and retaining a capacity of 608 mAh g^−1^ after 100 cycles [47].

Physical activation is considered a cost-effective method for activating carbon during thermal treatment. During pyrolysis, various degradation products such as CO_2_, CO, NH_3_, CH_4_, NO, water, and H_2_ are produced, depending on the initial source component. These products promote the formation of internal pores and extend or expand the range of pore sizes. Additionally, under slightly more intensive or concentrated conditions, external gas sources such as He, Ar, air, CO_2_, NH_3_, steam, or binary gas mixtures are utilized to achieve optimal internal pore volume or pore size. Physical activation facilitates the formation of micro-, meso-, and macropores, which typically require a comparatively higher temperature range of activation compared to the chemical activation process [48,49,50,51].

**Figure 7:**
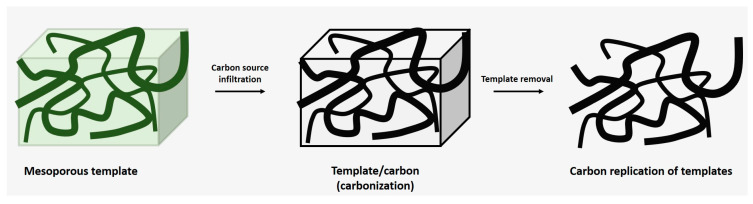
Schematic representation of the hard template strategy.

Hard template activation involves duplicating the molecular-level imprinting on the surface of the template structure at high temperatures (Figure 7). These templates can include zeolites, porous metals like Ni, Cu, and Fe, metal foams, metal powders, MOFs, silica, molten salts, or eutectic mixtures (high-temperature solvents). Hard templates induce stable mesoporous structures with large specific surface areas. However, there are disadvantages to removing hard solvents such as zeolites or silica-based templates, which require NaOH or HF, while metals like metal foam or powder necessitate acidic solutions. In the case of molten salts or eutectic solvent mixtures (e.g., LiCl/KCl, LiBr/KBr, LiI/KI), only water-washing steps are required for removal [52,53,54,55].

**Figure 8:**
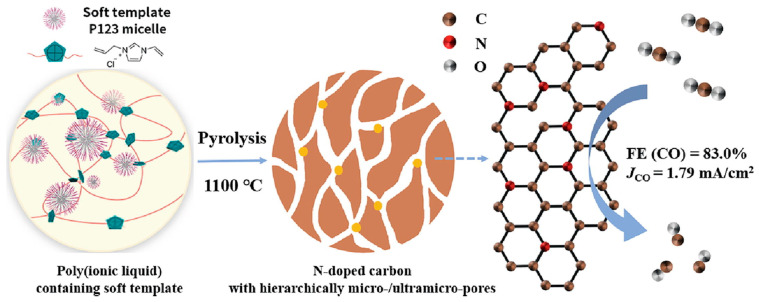
Synthetic pathway for porous poly (ionic liquid) CAV and the resulting carbon materials (adapted with permission from ref. [57], Copyright 2022 Elsevier).

Soft templating utilizes the supramolecular arrangement of carbon sources and block polymers, leading to the formation of a mesophase (Figure 8). The formation of this mesophase typically involves thorough catalytic or thermal treatment. It is crucial to consider the structural integrity of the soft template during thermal treatment or carbonization to ensure proper shaping of the resulting carbon products on its surface. As the soft template undergoes further carbonization, its presence gradually diminishes, serving as a source of gas templates and further inducing internal porosity in the targeted carbon products. The size, shape, and orientation of these supramolecular products or soft templates can be optimized through factors such as the solubility of components in the solution, polarizability, solution ratio, and other relevant parameters [46,56,57].

## 4. Carbon-Based Gel Materials for Energy Applications

### 4.1. Microbial Fuel Cell/Electrical Double-Layer Capacitance

In the utilization of fuel cell electrode materials within the benthic zone of the ocean, careful consideration must be given to the challenges posed by the unpredictable environmental conditions [58]. One notable application involves the utilization of a resorcinol–formaldehyde nitrogen–doped C foam electrode in microbial fuel cells (MFCs). However, traditional carbon foam materials face challenges in maintaining structural integrity under the high pressures found in the benthic zone, often resulting in a transition from a three-dimensional (3D) to a two-dimensional (2D) structure [59]. To address this issue, the Mungray research group developed carbon xerogels derived from resorcinol, urea, and formaldehyde aerogels through carbonization at 900 °C, specifically tailored for use in benthic microbial fuel cells (BMFCs) [60]. Additionally, magnetite was incorporated into the aerogel to produce magnetite-doped xerogel electrodes. This modification enhances the electrode’s suitability for colonization by bacterial communities, owing to its porosity, hydrophilicity, and biocompatibility. Furthermore, the incorporation of a stainless-steel base matrix ensures the structural stability of the 3D electrodes in highly pressurized benthic zones. Comparative studies demonstrate the superior performance of the xerogel electrodes, with a power density of 895 mW m^−3^, in contrast to the 14 mW m^−3^ exhibited by carbon felt electrodes. Notably, the periodic pattern of the carbon xerogel facilitates efficient electron transfer processes [60].

Madhav et al. synthesized carbon xerogel from resorcinol–formaldehyde polymer for application as an electrode in symmetric electric double-layer capacitors (EDLCs) [61]. They observed that the organic gel carbonized at 800 °C exhibited moderate textural properties, with a surface area of 791 m^2^ g^−1^, microarea of 177 m^2^ g^−1^, and mesoarea of 369 m^2^ g^−1^. Subsequent activation of the carbon xerogel with KOH, using a pelletized mixture of xerogel and KOH at a ratio of 1:6, resulted in enhanced textural properties, with a surface area of 1748 m^2^ g^−1^, microarea of 1164 m^2^ g^−1^, and mesoarea of 509 m^2^ g^−1^. This activation process facilitated K^+^ ion intercalation and oxidation (6KOH + 2C → 2K + 3H_2_ + 2K_2_CO_3_), leading to improved performance, with specific double-layer capacitance values of 186.1 F g^−1^ at a current density of 0.1 A g^−1^ and 168.5 F g^−1^ at 1 A g^−1^ [61]. It is important to note that the study did not differentiate the role of pelletized conditions in the fabrication of xerogel. While chemical activation undoubtedly enhances or improves the textural properties, the pelletized shape fabrication conditions may also play a role in the formation of micropores [41,42].

### 4.2. Supercapacitors

**Figure 9:**
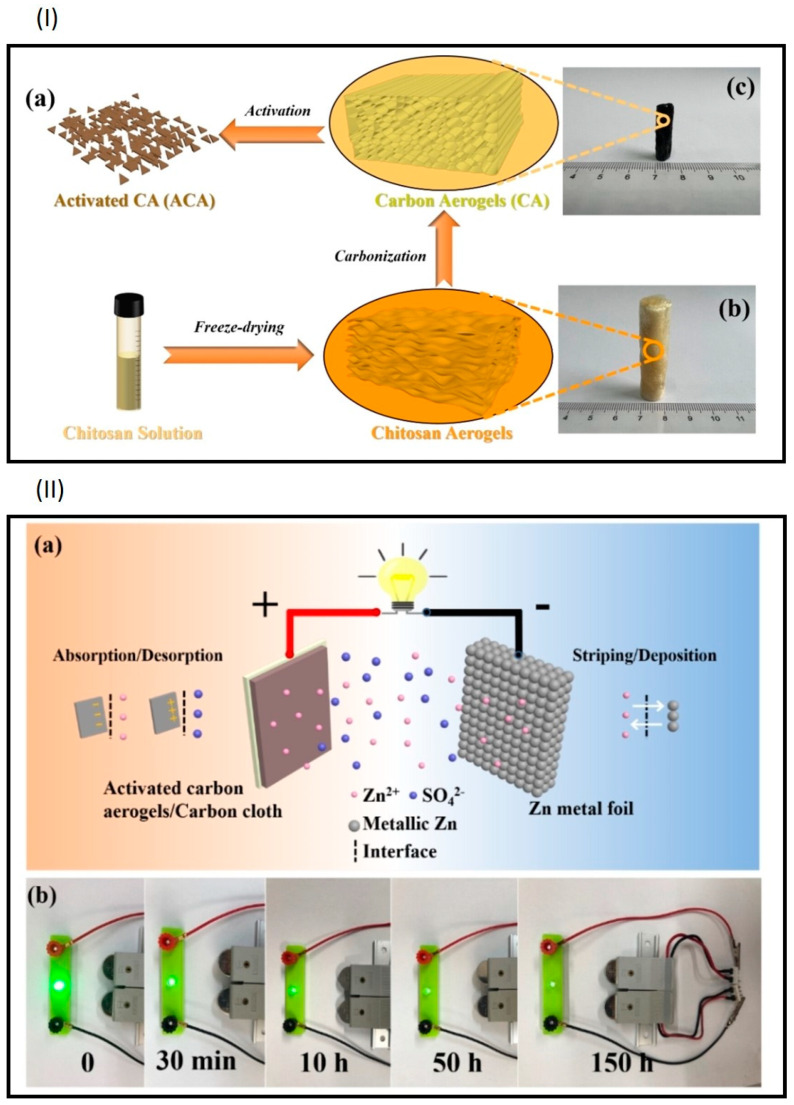
(**I**) (**a**) Schematic illustration for the fabrication of activated carbon aerogels (ACA), (**b**) chitosan aerogels, (**c**) carbon aerogels (**II**) (**a**) schematic illustration for the energy storage mechanism of the ZHC, (**b**) the application of our ZHC device to light up a 3 V LED. (Adapted with permission from ref. [63], Copyright 2023 Wiley).

Supercapacitors, relying on ion adsorption, inherently offer lower energy density compared to batteries based on faradic reactions. However, hybrid supercapacitors integrating battery type electrodes hold promise for surpassing the energy density of traditional supercapacitors and the power density of batteries [62]. The Liu group developed a Zn ion hybrid capacitors (ZHCs) utilizing a zinc anode and a carbon aerogel derived from chitosan as the cathode (Figure 9) [63]. The zinc anode offers high theoretical capacity (823 mAh g^−1^) and a low redox potential (–0.76V vs. SHE), enabling the attainment of high operating voltages [64,65]. Previous ZHCs employing carbon nanotubes (CNTs) as cathode material exhibited low faradic capacity (53 F g^−1^) due to their limited surface area (211 m^2^ g^−1^) [66]. In contrast, the Liu group employed a 3D porous interconnected carbon aerogel, which provides abundant active sites for ion/molecule adsorption and low-resistance pathways for ion/molecule transport, while also serving as an electrolyte reservoir. The carbon aerogels possess a surface area of 1000 m^2^ g^−1^ with predominantly low micropores and small-diameter mesopores. Utilizing KOH as the activating agent for the carbon aerogel led to the degradation of its structure into a 2D microsheet configuration with a significantly increased specific surface area (2267 m^2^ g^−1^) and the induction of numerous micro-/mesopores (Table 1). The ZHCs demonstrated a specific capacity of 299.5 F g^−1^ (133.1 mAh g^−1^) at a current density of 0.1 A g^−1^, yielding an energy density of 80 W kg^−1^ and exhibiting 86.3% capacity retention over 10,000 cycles at 2 A g^−1^ [63].

Raap et al. synthesized and compared graphene–doped carbon xerogel (GAG) and nitrogen–doped carbon xerogel (NAG), which exhibit analogous textural properties, with surface areas of approximately 1200 m^2^ g^−1^ and micropore volumes of approximately 0.50 cm^3^ g^−1^ [67]. GAG demonstrated a higher mesopore volume (0.48 cm^3^ g^−1^) compared to NAG (0.29 cm^3^ g^−1^). These materials were investigated for their application in supercapacitors. In a symmetric supercapacitor configuration, GAG^−^/GAG^+^ exhibited an improved specific capacitance of 19 F g^−1^ for the cell and 74 F g^−1^ for one electrode, along with an energy density of 6.6 mWh cm^−3^, compared to NAG^−^/NAG^+^ with specific capacitances of 12 F g^−1^ for the cell and 48 F g^−1^ for one electrode and an energy density of 4.2 mWh cm^−3^. It appears that the higher electrical conductivity of GAG contributes to its favorable performance, despite the increased wettability of the electrolyte due to nitrogen doping in NAG. The combination of higher electrical conductivity and a larger volume of mesopores facilitates ion mobility (such as Na^+^, OH^−^, and SO_4_^2−^) during the charge storage process, thus favoring enhanced performance [67].

### 4.3. Lithium Ion/Lithium Sulfur Batteries

**Figure 10:**
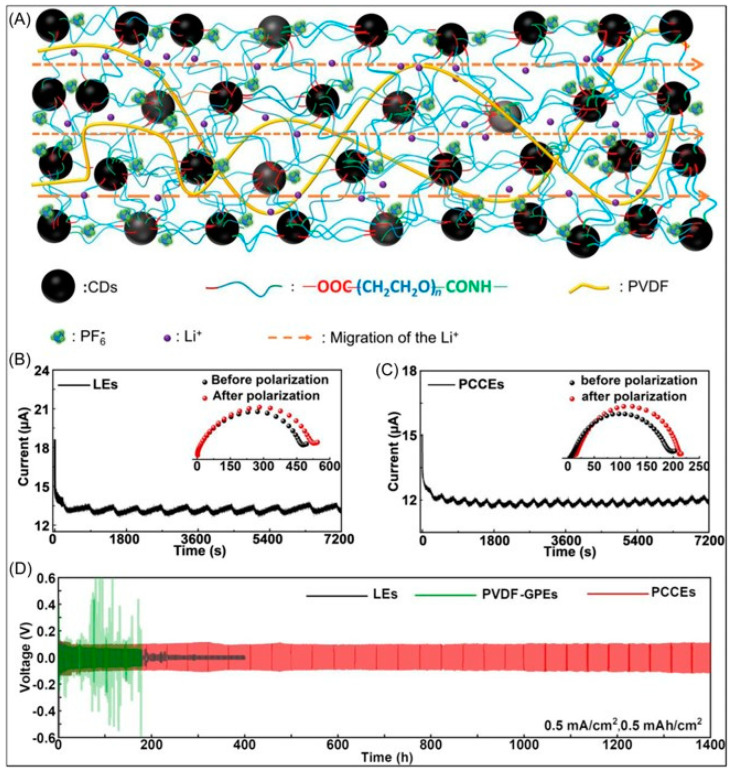
(**A**) Schematic illustrations detailing the operational mechanisms that enhance ion conductivity and ion transference number in polyethylene glycol–carbon dot composite electrolytes (PCCEs), (**B**) ion transference number evaluations performed on symmetric batteries with liquid electrolytes (LEs), (**C**) ion transference number evaluations using PCCEs as electrolytes, and (**D**) galvanostatic cycling tests conducted on Li/Li symmetric batteries with LEs, polyvinylidene fluoride–gel polymer electrolytes (PVDF–GPEs), and PCCEs, all under a constant current density of 0.5 mA cm^−2^ (adapted with permission from ref. [73], Copyright 2022 Wiley).

In lithium ion batteries, the implementation of solid-state or quasi-solid-state electrolytes has been pivotal in mitigating safety concerns associated with traditional liquid electrolytes. This paradigm shift has introduced two main categories: solid polymer electrolytes (SPEs) and inorganic solid electrolytes (ISEs), each characterized by distinct advantages and limitations. SPEs, lauded for their excellent processability and reduced interface impedance, nevertheless encounter a setback in ionic conductivity at ambient temperatures due to the crystalline phase formation impeding ion migration. Conversely, ISEs demonstrate superior ionic conductivity but suffer from processing limitations due to brittleness, resulting in compromised electrode interface integrity [68,69]. Gel polymer electrolytes (GPEs) serve as a promising compromise, amalgamating the merits of both SPEs and ISEs. To enhance GPE performance, various additives, such as boron nitride, have been incorporated, yielding improved mechanical properties. However, the integration of inorganic additives often yields heterogeneous phases within composite GPEs, hampering overall performance. To mitigate this issue, nanoparticles have been utilized, although their high surface reactivity predisposes them to unforeseen side reactions and agglomeration during battery operation, consequently diminishing battery capacities [70,71,72]. In a recent study by Huang et al., a novel approach involving the crosslinking of polyethylene glycol (PEG) and carbon dots (CDs) gel polymer, alongside the incorporation of polyvinylidene fluoride (PVDF) as an electrolyte, exhibited remarkable outcomes (Figure 10) [72]. This approach yielded an impressive ionic conductivity of 5.5 mS cm^−1^ and an ion transference number of 0.71 at room temperature. Notably, PEG hindered CD agglomeration, while CDs facilitated the dissociation of lithium salts and reduced the crystallinity of the polymer host, consequently enhancing ionic conductivity and reducing concentration polarization within the battery, thus promoting Li^+^ migration [73].

**Figure 11:**
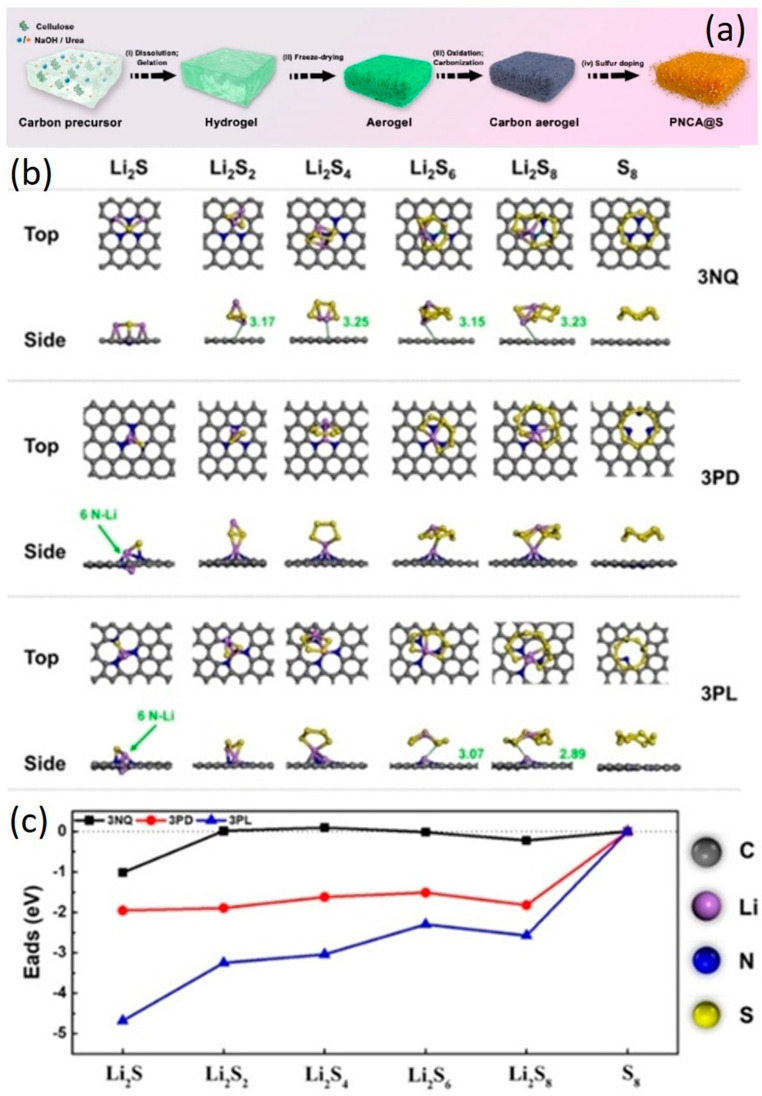
(**a**) Schematic of the preparation of cellulose–based N–doped carbon aerogels, (**b**) adsorption models (top/side view), and (**c**) adsorption energies of different polysulfides on different N–doped configurations. Note: Green values indicate bond lengths; in 3PL/3PD_Li_2_S_6_, Li@N is the most stable bond (adapted with permission from ref. [76], Copyright 2021 Wiley).

Li–S batteries are considered one of the most promising alternative energy storage devices due to their high theoretical energy density of 2600 Wh kg^−1^ (2800 Wh L^−1^). However, they face technical challenges such as shuttle effects and volume expansion of sulfur, leading to rapid decay, capacity loss, low coulombic efficiency, and poor rate performance. During discharge, long-chain polysulfides are formed in the cathode, which are soluble in the electrolyte. These soluble polysulfides diffuse through the separator to the anode (Li metal) and are converted to Li_2_S_2_ or Li_2_S, which are insoluble and passivate the anode. The deposition of these insoluble compounds hinders electrical conductivity, ion conductivity, and results in the loss of active materials. Functional groups based on nitrogen (pyridinic, pyrrolic, graphitic/quaternary) play a crucial role in the adsorption of polysulfides, thereby reducing shuttle effects [11,74,75]. The strong dipole–dipole interaction between polysulfides and the Li–S cathode originates from the pyridinic nitrogen. Understanding the role of nitrogen-containing groups in carbon materials is essential for designing strategies to mitigate the shuttle effects in Li–S batteries. Yuan’s group fabricated a pyrrolic-N-doped carbon aerogel from cellulose, which features a 3D honeycomb-like structure (Figure 11) [76]. They evaluated the catalytic conversion mechanism of polysulfides, demonstrating its potential in reducing the shuttle effect and enhancing the performance of Li–S batteries. A high–pyrrolic–N–content carbon aerogel, characterized by XPS deconvolution as containing 54.6% pyrrolic-N, 11.6% pyridinic–N, 21.4% quaternary–N, and 12.3% nitrate, demonstrated a capacity of 1249 mAh g^−1^ at 0.2 C. DFT analysis reveals that pyrrolic–N exhibits the strongest stability across different polysulfide phases and the highest adsorption capacity, which likely inhibits shuttle effects most effectively. In contrast, graphitic–N is less stable compared to pyrrolic– and pyridinic–N. Additionally, the interaction of pyrrolic-N with polysulfides is significantly greater than that of pyridinic–N, indicating that pyrrolic–N groups play a more crucial role in the adsorption and catalytic conversion of polysulfides.

### 4.4. Al Batteries

Aluminum (Al) batteries are considered a promising alternative energy storage technology. One of the key challenges is managing fluctuating electricity and achieving fast charging, which are crucial for uniform current distribution on the electrode surface and rapid ion/electron transport [77,78,79]. Conventional aluminum foil, used as both active material and current collector, suffers from non-uniform current distribution and current density fluctuations, leading to dendrite growth during charge–discharge cycles, a major safety concern [80,81]. The use of ionic liquid electrolytes exacerbates these issues by corroding or pulverizing the Al foil and increasing the severity of current density fluctuations [82,83].

The 2D planar structure of Al foil as anode and cathode materials, such as graphite paper, limits the adaptability of Al batteries due to significant fluctuations in current densities. Planar cathode materials face high local current densities and non-uniform, slow ion/electron transport during charge–discharge cycles. The continuous intercalation and deintercalation of ${AlCl}_{4}^{-}$ ions in planar, compact graphite paper cause structural expansion and collapse at high current density fluctuations [84,85,86]. Additionally, loading active materials such as graphite, graphene, or Cu metal–organic frameworks with binders and conductive materials on planar current collectors increases the ineffective mass load of the cathodes and impedes ion/electron transport at high current densities [87,88].

**Figure 12:**
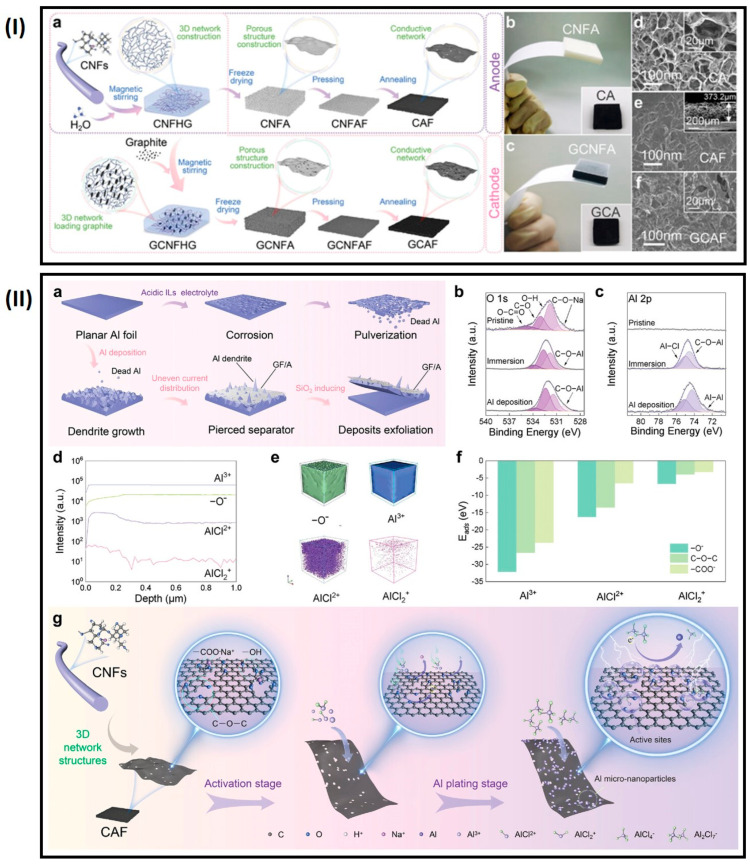
(**I**) (**a**) Synthesis schematic of 3D porous carbon aerogel film (CAF) and graphite composite carbon aerogel film (GCAF), photos of (**b**) cellulose nanofiber aerogels (CNFA) (CA, inset), and (**c**) graphite composite CNFA (GCNFA) (GCA, inset), and SEM images of (**d**) CA, (**e**) CAF, and (**f**) GCAF; (**II**) (**a**) Issues with planar Al foil anode during charge/discharge, XPS spectra of CAF and activated CAF in electrolyte with Al deposition at 1 mA cm^−2^: (**b**) O 1s and (**c**) Al 2p, (**d**) depth profiles, (**e**) 3D sputtered volume images of AlCl_3__−x_^x^^+^ ions on CAF electrode immersed in electrolyte (TOF-SIMS), (**f**) DFT-calculated adsorption energy of O–containing functional groups to AlCl_3__−x_^x^^+^ ions, and (**g**) schematic showing Al–induced deposition mechanism on CAF anode with O–containing groups (adapted with permission from ref. [89], Copyright 2023 Wiley).

Adhesion between the current collector and active materials must be maintained at low levels (0.7–3 mg cm^−2^), presenting a fundamental obstacle to improving the high energy density of Al storage batteries [83,87]. To address these challenges, the Jiao group fabricated a hierarchical 3D carbon aerogel film (CAF) for the anode and integrated it with a graphite composite carbon aerogel film (GCAF) as the cathode (Figure 12) [89]. 

The oxygen-containing functional groups on the CAF anode facilitate uniform current distribution, while the GCAF cathode supports a high effective mass loading (9.5–10 mg cm^−2^), enhancing utilization efficiency. The lightweight CAF‖GCAF full battery demonstrated a discharge capacity of 115.6 mAh g^−1^ after 200 cycles and achieved a shorter charging time of 7 min at a current density of 1000 mA g^−1^ [89]. In this study, CA and CAF serve as host matrices with 3D porous structures that favor electron and ion transport. This configuration effectively regulates the nucleation of Al metal, potentially suppressing dendritic growth during charge–discharge cycles. Additionally, the hierarchical porous structure of CA and CAF, when used as current collectors in the cathode, supports high graphitic mass loading, resulting in enhanced capacity. The elimination of binders and conductive agents in the lightweight integrated carbon aerogel contributes to high energy density. In acidic electrolyte solutions, these structures exhibit anti-corrosion properties. The folded, staggered channels within the CAF and GCAF shorten ion diffusion distances inside the electrode. The hierarchical structure with a large specific surface area provides ample space for Al deposition and the intercalation of ${AlCl}_{4}^{-}$. Abundant oxygen-containing functional groups facilitate uniform deposition sites for Al, reducing dendrite growth. These functional groups also improve the wettability of the ionic liquid electrolyte on the 3D hierarchical porous structure, promoting fast ion transport within the electrode and adapting to large fluctuations in current densities [89].

### 4.5. Zn–Air Batteries

**Figure 13:**
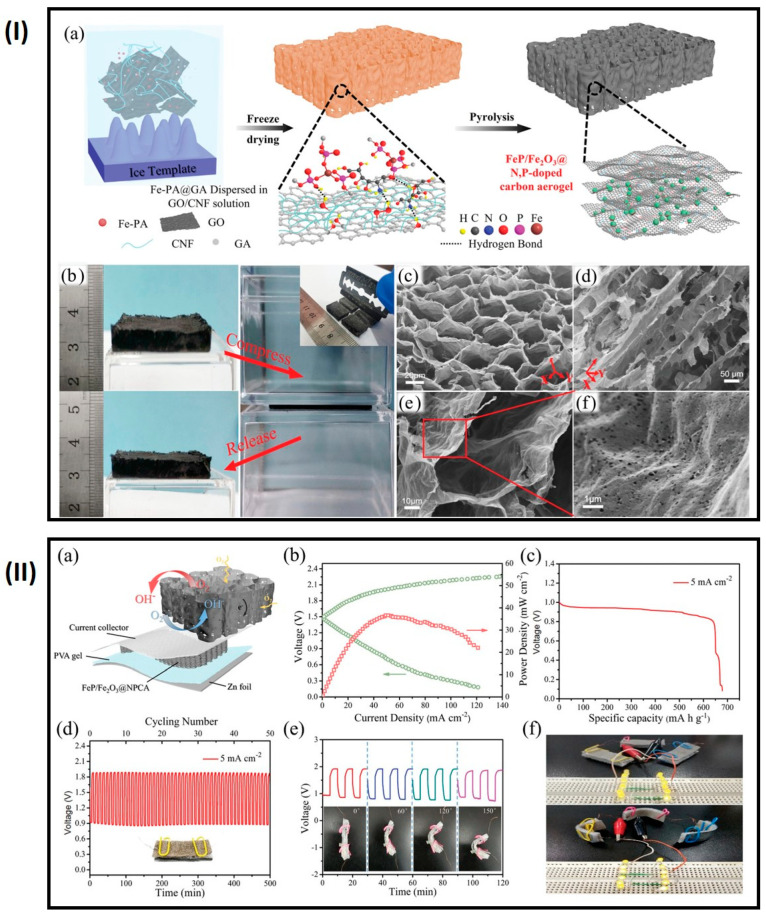
(**I**) (**a**) Schematic of fabrication, (**b**) image of the compressibility, and (**c**–**f**) SEM image of FeP/Fe_2_O_3_@NPCA carbon aerogel; (**II**) (**a**) scheme of ZABs, (**b**) galvanostatic cycling with potential limitations (GCPL) profile, (**c**) specific capacity at 5 mA cm^−2^, (**d**) cyclic stability at 5 mA cm^−2^, (**e**) cyclic stability under different bends at 5 mA cm^−2^, and (**f**) image of LED illuminations by ZABs (adapted with permission from ref. [93], Copyright 2020 Wiley).

Freestanding air electrodes are considered potential candidates for the development of wearable, flexible energy storage devices, such as Zn–air batteries (ZABs). ZABs offer a high theoretical energy density (1086 Wh kg^−1^) and are cost-effective, environmentally friendly, and safe for use in flexible wearable devices [90,91,92]. However, freestanding electrodes like carbon cloth, with their compact fibrous structures, are inefficient in terms of air/electrolyte permeability and diffusion during electrochemical operation. The loading of active materials on these compact fibrous materials is limited due to the small surface area of carbon cloth [93,94]. To address these challenges, the Lu group developed a 3D honeycomb nanostructured carbon aerogel with N, P doping and in situ growth of FeP/Fe_2_O_3_ nanoparticles as a bifunctional air cathode using a directional freeze-casting process (Figure 13) [93]. Although precious metal or metal oxide electrocatalysts such as Pt/C, Ir/C, and RuO_2_ are commonly used to counter sluggish kinetics, large overpotentials, and poor energy and power densities of the oxygen evolution reaction (OER) and oxygen reduction reaction (ORR), their high cost limits their application in flexible devices [95,96]. The fabricated 3D-structured carbon aerogel provides mechanical stability for bending and compression, as well as efficient gas/electrolyte diffusion and ionic conductivity. The developed freestanding air cathode for ZABs delivers a specific capacity of 676 mAh g^−1^ and an energy density of 517 Wh kg^−1^ at 5 mA cm^−2^. Heteroatom doping with N and P enhances electrocatalyst performance: pyridinic-N improves the onset potential, graphitic N regulates the diffusion-limiting current of ORR, and F–N groups favor the ORR process. P atoms influence the charge distribution and geometric symmetry of adjacent C atoms, facilitating accelerated ORR by accepting electron pairs on O_2_. N and P doping in the carbon induces defects and active sites, providing highly surface-active sites, facilitating ion/charge transport, and improving OER/ORR performance in the 3D porous network.

It is essential to note that uniform heteroatom doping (e.g., N, O, S, B, P) is critical in carbon aerogels. Heteroatom doping can influence the surface area, pore volume, and mechanical strength of the aerogels. More importantly, uniform distribution of these dopants is crucial, as uneven doping can cause localized variations in conductivity. For consistent catalytic sites and efficient electrochemical performance, it is imperative that heteroatom doping remains consistent and uniform [97,98]. Energy density (Wh kg^−1^) refers to the amount of energy stored or available in a given mass of an energy system. Conversely, power density (W kg^−1^) describes the rate at which this stored energy can be delivered or converted. To increase power density, efficient electron transfer within the electrode materials is essential, as it leads to higher power density. Enhancing ion mobility facilitates faster charge transfer kinetics, which is also crucial for achieving higher power density. A hierarchical 3D structure is effective in reducing ion diffusion pathways, thereby promoting faster charge/discharge kinetics. In this context, it is important to consider the degree of polyaromatic condensation, the R-factor, and the crystalline phase of carbon materials. These factors can enhance the electrical conductivity and structural integrity of carbon materials, supporting rapid charge/discharge cycles. Additionally, the hierarchical structure of the material, including the pore size distribution (ultra-, super-, micro-, meso-, and macropores), plays a significant role. For energy density, a larger surface area effectively accommodates the adsorption and desorption of ions, thereby increasing energy density. The energy storage mechanism can involve ion intercalation, pore filling, surface adsorption, and defect binding. Given the trade-off between energy density and power density, the crystallinity and hierarchical structure of the material are fundamental factors to balance these attributes. All these factors depend on the specific carbon materials and their mechanisms for efficient ion storage, whether through intercalation, pore filling, or surface adsorption. High crystallinity enhances intercalation capacity, while a hierarchical structure supports pore filling and smooth diffusion. Therefore, optimizing pore ranges is essential to achieving substantial energy density and power density [9,10,99]. 

## 5. Stability of Carbon Materials

**Figure 14:**
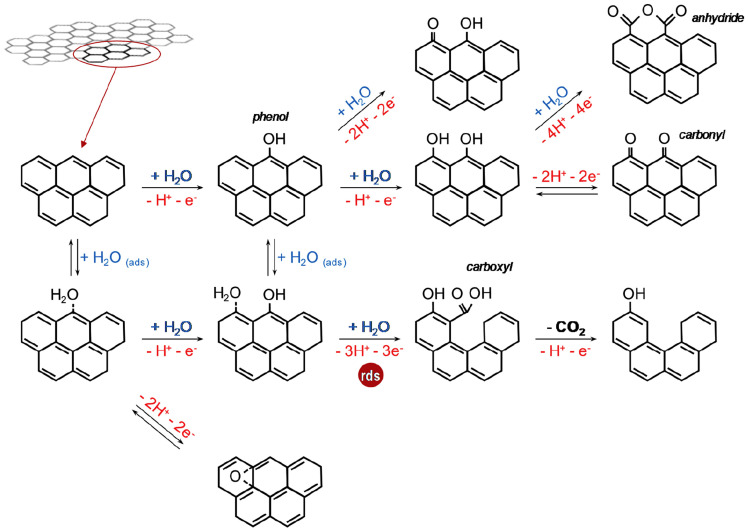
Scheme of carbon electro–oxidation mechanism (adapted with permission from ref. [105], Copyright 2019 Elsevier).

Carbon-based materials offer a cost-effective solution for various energy storage and conversion applications, primarily due to their environmentally friendly nature, abundances, and synthesis flexibility [41,42]. However, in electrochemical devices, carbon-based materials are susceptible to electro-oxidation at high positive potentials, leading to carbon oxidation reactions (CORs) and the formation of carbon dioxide (CO_2_) at approximately 0.207 V vs. the normal hydrogen electrode (NHE) at 25 °C [100]. In electrochemistry, carbon corrosion poses a significant challenge to achieving stable operation, crucial for understanding oxidation kinetics and carbon electrode degradation. Despite ongoing research, the precise factors contributing to degradation remain unclear, including surface area, interlayer spacing of the graphitic layer, the percentage of graphitic domains, and the presence of oxygen-containing groups [101,102]. Various strategies have been proposed to address this issue, such as modifying surface functional composition or controlling textural properties. However, the specific contributions of each approach remain poorly defined [103,104]. The Lazaro research group sought to elucidate the electrochemical oxidation of carbon materials through empirical investigation, focusing on carbon xerogels with varying surface chemistries and structures [105]. Their study involved electro-oxidation experiments conducted at 1.2 V vs. RHE using carbon xerogel electrodes. Results indicated that the presence of –C–O functional groups and a higher degree of ordering in the carbon structure conferred resistance to carbon oxidation (Figure 14).

The COR process involves a sequence of parallel and series reactions. Initially, the formation of phenol-like groups takes place, followed by the subsequent generation of carboxylic groups through the oxidation of the phenolic group by adjacent adsorbed water molecules on carbon atoms. Subsequently, complete oxidation to CO_2_ occurs from the carboxylic groups, which exhibit greater reactivity compared to other oxygen-containing functional groups [106]. Water molecules also facilitate the formation of oxygen-containing groups, such as carbonyls/quinones (C=O), which can reversibly form hydroquinone or irreversibly form anhydrides. Ultimately, the presence of oxygen-containing functional groups, such as phenol, impedes the electro-oxidation of carbon, as it results in the formation of stable oxygen-containing groups (carbonyl/quinone) that do not undergo further oxidation to CO_2_. When considering microstructural aspects, edge carbon atoms are more susceptible to electrochemical oxidation compared to basal atoms. Moreover, from a textural perspective, wider pores and the presence of carboxylic groups contribute to the carbon–electrolyte interface, thereby promoting electro-oxidation by water. The surface nanostructure exerts an influence on the oxidation rate. Higher degrees of disordering (I_D_/I_G_ ratio) render the material more susceptible to corrosion, and this ratio can serve as a useful parameter for assessing the electro-oxidative properties of carbon xerogels [105,107].

The formation of three-dimensional carbon aerogels is driven by physical interactions such as electrostatic interactions, hydrogen bonding, and π–π interactions. These interactions can lead to irreversible deformation of the porous structure, compromising its mechanical and structural integrity. However, incorporating multiple structural units through double-network and multinetwork skeletons can enhance the mechanical stability, compressive resilience, and elastic properties of the aerogels. This approach also improves mechanical strength through chemical crosslinking, resulting in a layered porous structure with an orderly arrangement of oriented carbon aerogels [108,109]. Wang’s group demonstrated this by preparing graphene aerogels with intertwined tubular carbon nanofibers (TCNFs) using oriented freeze drying [110]. The chemical crosslinking between graphene oxide and poly(oxypropylene) diamines (D_400_) forms hybrid aerogels, which exhibit mechanical compressibility of up to 80% and can withstand 50 compression/release cycles.

Carbon-based materials generally exhibit high thermal stability, maintaining their structural integrity and porosity even at temperatures exceeding 1000 °C. Oxygen-containing surface functional groups begin to desorb from the carbon surface at temperatures above 300 °C. Temperature-programmed desorption analysis indicates that hydroxyl groups desorb at 120–200 °C, while carboxylic groups desorb above 200 °C. Despite these desorption events, the structural and textural integrity of the material is preserved [111,112,113].

## 6. Sustainable Approach

Biomass resources such as trees, plants, leaves, fruit peels, grasses, agricultural waste (e.g., rice husk), tree bark, and nutshells serve as alternative sources for carbon product synthesis. These biomass resources not only offer value-added carbon products from low-cost, abundant material resources but also provide natural molecularly assembled soft templates. This allows for numerous opportunities to modify or tune properties due to the varying atomic ratios of heteroatoms and different gas template sources, which influence the surface chemistry and textural properties of the carbon products [114]. Utilizing such biomass reduces dependency on fossil fuels and repurposes waste materials into value-added products. Additionally, carbon aerogels derived from biomass are renewable and biodegradable; when disposed of in the environment, they gradually break down through biological processes [115].

Reproducibility of similar properties across different batches is a significant concern due to variations in synthesis conditions, which can lead to differences in textural and surface properties, ultimately affecting performance in applications. Many studies fail to identify, document, or report all conditions throughout the process, from the source to the final product preparation and storage. From a fundamental perspective, materials engineering at the molecular level requires careful consideration of every possible aspect. Initially, it is essential to check, report, and document all fundamental properties of the source materials. During synthesis and carbonization, every small aspect must be meticulously checked, reported, and understood to achieve consistent quality. In the synthesis process, it is crucial to consider the properties of the source materials, the ratio of reactants, temperature, pressure, stirring rate, reactor volume, height, radius, reaction medium (e.g., air, N_2_), purity, reaction time frame, sequence and timing of reactant addition, physical properties of the stirrer, environmental conditions of the synthesis room, equipment validation, and proper management of synthesis apparatus (e.g., cleaning and storage conditions). For carbonization, factors such as amounts, container size and shape, loading height, heating rate, duration, type of medium, flow rate, carbonization area, placement of products in the furnace, and sealing conditions must be considered. Even aspects that may seem insignificant should be taken into account, as unexpected findings or insights could arise from thorough examination.

## 7. Technology Readiness Levels (TRLs)

**Figure 15:**
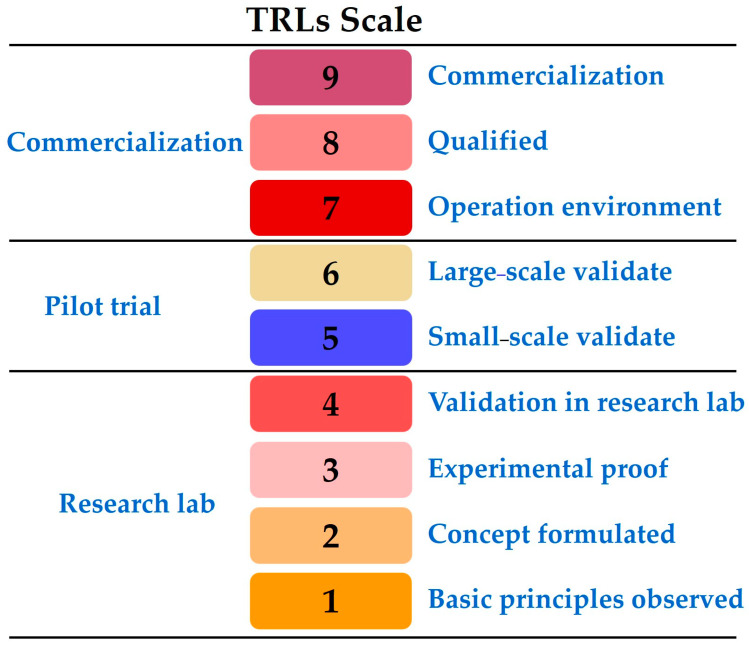
Schematic of the Technology Readiness Level (TRL) scale.

Technology readiness levels (TRLs) categorize, evaluate, monitor, and validate the progress of technologies in their respective fields of research (Figure 15) [40]. The production of carbon aerogel has reached a maturity level that is high enough for it to be commercialized and supplied to various demand areas although substantial research is still being carried out on properties development or cost reduction phenomena in synthesis or processing. Numerous aerogels are viable at a commercial scale in different applications. Carbon aerogel (molecular weight 12.01 g mol^−1^) of Aerogel Technologies, LLC, graphene aerogel (density 12.5 mg cm^−3^) of Graphene Laboratories Inc., and carbon aerogel–silicon hybrid by Aspen Aerogels are commercially available for applications in batteries, supercapacitors, and electrochemical sensors. 

From a cost reduction perspective, the emphasis should be on utilizing readily available, naturally derived precursors such as cellulose, chitosan, and starch to formulate gel-based materials. Optimizing synthesis routes and fabrication processes, including drying, temperature, and pressure parameters, can minimize unnecessary energy consumption. Ultimately, efficient performance based on material properties and longevity is crucial for cost-effectiveness. From a technoeconomic standpoint, employing highly efficient materials can contribute to cost reduction strategies, even if this necessitates adopting comparatively expensive processes.

**Table 1:** Performance comparison of carbon aerogel materials for different energy devices.

Devices	Materials	Performances	Ref.
Li–S batteries	*C-aerogel	Capacities 1290 mAh g^−1^ (1st cycle)	[47]
Li–S	*C-aerogel	Capacity 1249 mAh g^−1^ (0.2 C)	[76]
*BMFC	*C-xerogel	Power density 895 mW m^−3^	[60]
*BMFC	*C-felt	Power density 14 mW m^−3^	[60]
*EDLC	Resorcinol–formaldehyde/KOH (activation)	Specific double-layer capacitance values 186.1 F g^−1^ at current density 0.1 A g^−1^ and 168.5 F g^−1^ at 1 A g^−1^	[61]
Supercapacitor	Graphene-doped *C xerogel (GAG^−^/GAG^+^)	Energy density of 6.6 mWh cm^−3^, power density 165 W kg^−1^	[67]
Li ion batteries	Polyethylene glycol–carbon dot composite electrolytes	Ionic conductivity 5.5 mS cm^−1^, ion transference number 0.71	[73]
Li–S batteries	Glucose/ZnCl_2_ C gel	Initial capacity of 1290 mAh g^−1^, capacity 608 mAh g^−1^ (100 cycles)	[47]
Zn ion capacitor	Chitosan aerogel	Energy density 106.5 Wh Kg^−1^, power density 3108 W Kg^−1^	[63]
Al batteries	*C aerogel film/graphite composite *C aerogel film	Discharge capacity 115.6 mAh g^−1^ after 2000 cycles	[89]
Zn–air batteries	*C aerogel (N, P doped)/FeP/Fe_2_O_3_	Specific capacity 676 mAh g^−1^, energy density 517 Wh kg^−1^ at 5 mA cm^−2^, overpotential 402 mV at 10 mA cm^−2^, Tafel slope 86 mV dec^−1^	[93]
*SSBs	N-, S-co-doped C aerogel	Reversible capacity 788 mAh g^−1^ at 0.1 C	[116]
Potassium supercapacitor	O, N, B–*C aerogel	Energy density51.8 Wh kg^−1^, power density 443 W kg^−1^	[117]
OER	Fe, N–*C aerogel	Onset potential 0.9 V, half-potential 0.7 C (alkaline); onset potential 0.8 V, half-potential 0.5 C (acidic)	[118]

*C: carbon; *BMFC: benthic microbial fuel cell; *EDLC: electric double-layer capacitance; *SSBs: sodium–sulfur batteries.

## 8. Summary and Future Research Perspectives

Carbon gel-based energy materials present significant opportunities for optimizing carbon precursors and varying chemical synthesis designs, leading to improved microstructure and pore properties, including void volumes, pore size, and distribution, compared to traditional carbon-based materials. However, supercritical drying poses a challenge for scaling up the production of gel-based materials to industrial levels due to its uneconomical and unsafe nature. Thus, developing ambient drying conditions is imperative. In energy devices, the higher electrical conductivity of carbon-based materials enhances performance. While surface functional groups or heteroatom doping can improve the wettability of electrolytes, it is crucial to balance this with the potential compromise in electrical conductivity and the degree of polyaromatic condensation during carbonization. This balance affects the microstructure and lifespan of carbon materials, making it essential to evaluate the trade-offs between heteroatom doping and electrical conductivity. Mesophase structures facilitate ion mobility, necessitating the determination of the optimal mesopore size range for efficient mobility. In Li–S batteries, various nitrogen configurations (pyrrolic, pyridinic, graphitic) in the carbon structure help reduce shuttling effects. Pyrrolic nitrogen, in particular, plays a crucial role in the adsorption of polysulfides and catalytic conversion. Future research should focus on determining the optimal content percentages of these configurations for efficient catalytic performance.

The microstructure of carbon-based aerogels, including the degree of aromatic condensation, crystallite size, lateral size, and R-factor, significantly impacts electrochemical properties, structural integrity, and overall performance. However, there has been limited research in this area. Establishing correlations between these microstructural properties and the mechanistic performance of energy materials is necessary. Adhesion between active materials and current collectors is another critical area requiring further research. Achieving proper adhesion with a low level of carbon active materials is challenging, as it conflicts with the strategy to reduce binder content and increase active mass loading. Addressing these issues is vital for the advancement of carbon aerogel strategies. Additionally, O-containing functional groups are susceptible to electro-oxidation, leading to material corrosion. Understanding the role of these functional groups in electro-oxidation is essential to maintaining the structural integrity of carbon aerogels. Future research should aim to evaluate and mitigate the impact of electro-oxidation on these materials. By addressing these research directives, the development and application of carbon gel-based energy materials can be significantly advanced, leading to more efficient and durable energy storage solutions.

## Data Availability

Not applicable.
